# Molecular Diversity of Microbes Associated with Fermented Bamboo Shoots

**DOI:** 10.21315/tlsr2022.33.3.9

**Published:** 2022-09-30

**Authors:** Vijay Kumar, Bindu Naik, Sachin Sharma, Akhilesh Kumar, Javed Masood Khan, Mohammad Irfan

**Affiliations:** 1 Himalayan School of Biosciences, Swami Rama Himalayan University, Swami Rama Nagar, Jolly Grant, Dehradun, Uttarakhand 248140 India; 2 Department of Life Sciences, Food Technology, Graphic Era (Deemed to be University), Bell Road, Clement Town, Dehradun, Uttarakhand 248002 India; 3 Department of Food Technology, Doon (P.G.) College of Agriculture Science and Technology, Selaqui, Dehradun, Uttarakhand 248011 India; 4 Department of Food Science and Nutrition, Faculty of Food and Agricultural Sciences, King Saud University, 2460, Riyadh 11451, Saudi Arabia; 5 Plant Biology Section, School of Integrative Plant Science, Cornell University, Ithaca, New York, USA

**Keywords:** Probiotic Bacteria, Fermented Bamboo Shoots, *Bacillus* Species, Antibacterial Activity

## Abstract

Fermented bamboo shoots are rich in high protein, carbohydrates, fibre and minerals while low in fat content. In the North-East region of India and other Asian countries, they are mostly used in various food preparations. The present study was undertaken to explore the diversity of bacteria associated with Bamboo shoots and to evaluate their antibacterial profile. Based on the results the fermented bamboo shoots showed viable counts ranging from 6.55 ± 0.91 log CFU/g to 7.86 ± 1.21 log CFU/g. The 16s rRNA sequence analysis showed that these isolates belonged to the genus *Bacillus* (*Bacillus safensis, B. tequilensis, B. siamensis, B. nakamurai, B. subtilis)* and Enterobacter. These isolates have not been reported previously from fermented bamboo shoots except *B. subtilis*. Surprisingly, no *Lactobacillus* species or molds were found in any of the samples tested. Potent antibacterial activity was recorded against *Klebsiella*, *Staphylococcus aureus*, *Salmonella* and *B. cereus*.

**Highlights**
- Fermented bamboo shoots are source of *Bacillus safensis, B. tequilensis, B. siamensis, B. nakamurai, B. subtilis* and *Enterobacter*.
- These isolates have not been reported previously from fermented bamboo shoots except *B. subtilis and Enterobacter*.
- Interestingly, no *Lactobacillus* species and molds were not detected in any of the analysed samples.
- Potent antibacterial activity was recorded against *Klebsiella*, *Staphylococcus aureus*, *Salmonella* and *B. cereus*.

## INTRODUCTION

Probiotics are microbial cell components or microbial cell preparations that have a favourable influence on the host’s health and well-being. They positively influence gastrointestinal infections, hold antimicrobial activity, vitalise the immune system, anti-mutagenic activity, anti-carcinogenic properties, anti-diarrhea activity, etc. Probiotics are associated with the digestive tract of humans and animals. Beneficial strains, which can be used as probiotic sources, are mostly found in the genera *Bifidobacterium* and *Lactobacillus*, and some of these strains have anti-inflammatory qualities (Isolauri *et al*. 2004; Behera & Balaji 2021). Fermentation of foods is usually controlled action of micro-organisms to alter the texture of food, upgrade their quality, preserve and produce characteristic flavour and aromas and have additional advantages of giving bio-supplements and minerals. Fermented food holds numerous microorganisms from the genera *Lactobacillus*, *Bifidobacterium*, *Saccharomyces*, *Enterococcus*, *Streptococcus*, *Pediococcus*, *Leuconostoc* and *Bacillus* and has been shown to have health advantages. The human microbiome is gaining a lot of attention these days, and studies have already shown that changing it can have far-reaching consequences. Despite preservation, fermented foods can likewise have the additional advantages of achieving flavour, expanded edibility, enhancing nutritious esteem and pharmacological qualities. Each fermented food is connected to an exceptional consortium of micro-flora which builds the levels of protein, vitamins and basic amino acids (Melini *et al*. 2019). Bamboos, sometimes known as the green gold, are a genus of giant woody grasses that belong to the Poaceae family and subfamily Bambusoideae. They are well-known for their environmental benefits (Goyal *et al*. 2010). Bamboo shoots, on the other hand, have yet to be fully utilised as a food source. Fermented bamboo shoots are a typical kind of food in north Indian cooking, particularly in Manipur, Nagaland and Sikkim. It is also used as food in other parts of the world. They are well-chosen healthiest foods because of their high protein, carbohydrate, vitamin, fibre and mineral content and low-fat content. It promotes health benefits such as antioxidant activity, cholesterol-lowering activity, anticancer activity, acts as an immune booster, anti-aging, prevents cardiovascular disease, is helpful in weight loss, decreases blood pressure, is rich in flavonoids, glycosides, and is rich in probiotics (Thakur *et al*. 2016; Behera & Balaji 2021). Bamboo shoots, whether fresh or fermented, are one of the most popular traditional foods among various ethnic groups. *Ushoi, soibum, rep, mesu, eup, ekhung, hirring* and other bamboo-based traditional meals are among the most popular (Choudhury *et al*. 2012). The fermented bamboo shoots are the hub of various beneficial microorganisms which may be of indigenous origin, starters, or may enter from utensils, containers, earthen pots that biochemically and organoleptically modify substrate into edible products. The type of microorganisms present during the fermentation is responsible for the change in the chemical components of the raw materials, enhancing nutritional value and providing health benefits (Tamang 2010; Tamang *et al*. 2016). In this context, the present study was undertaken to explore the molecular diversity of microbes associated with fermented bamboo shoots and evaluation of the antimicrobial potential of these isolates against pathogenic microbes.

## MATERIALS AND METHODS

### Sample Collection and Microbiological Analysis of Probiotic Bacteria from Bamboo Shoots

In January and February 2018, 10 samples of fermented bamboo, as well as raw bamboo samples weighing about 300 g each, were obtained from various locations throughout Manipur. Total viable counts were determined after selective isolation and microbiological examination. The samples were vortexed and serially diluted in sterile normal saline (0.85% NaCl (w/v) before plating into triplicate plates on MRS agar media (HiMedia, India) for the total viable count and selective isolation. The colony-forming unit (CFU) was determined after 24 h at 37°C. Potato dextrose agar media was used for the isolation of yeasts and molds.

Based on the colony colour, shape, size and appearance, and cell morphology (cocci, bacilli, cell arrangement, etc.), the isolates were selected (Tindall *et al*. 2010). Preliminary identification of isolates was done by colony morphology and Gram staining. The purity of the isolates was checked, and purified isolates were preserved in glycerol (20% w/v).

### Proximate Analysis of Raw and Fermented Bamboo Shoot

The raw and fermented bamboo shoots were collected from the local people. The procedure described by these people was used for fermentation. The bamboo shoots were sliced and dried in the sun for 10 to 15 min before being placed in an earthen pot to ferment for two months (25°C–27°C; room temperature) with the addition of a tiny amount of water and salt. The shoots then are taken out and dried in the sun until they are about 50% dry. The dried pieces are then stored and combined in a cane/bamboo basket for additional drying. The moisture content was determined by the hot air oven method (AOAC 1990, no. 947.05). The total phenolic in the sample was calculated using the Folin-Ciocalteu reagent (Bray & Thorpe 1954). Ten grams of sample was homogenised in 90 mL of sterile physiological saline (0.85%, w/v) and pH was determined using a pH meter (Eutech-700, Thermo Scientific, India). A pH meter calibrated with standard buffer solution was used to determine the pH. Ash content was determined by dry ashing in a muffle furnace at 600°C until white grayish ash was obtained (AOAC 1990). Titratable acidity was estimated according to the standard method (AOAC 1990). The filtrate of homogenate was titrated with 0.1 N NaOH to an endpoint of phenolphthalein (0.1% w/v in 95% ethanol). Reducing and total sugars were determined by the method of Lane and Eynon (Gandhi *et al*. 2017).

### Molecular Characterisation

Selected isolates (based on colony colour, size and shape on MRS agar media and micromorphology such as cocci, bacilli, cell arrangement, etc.) were characterised using a molecular approach based on 16S rRNA gene analysis. Genomic DNA was isolated as described previously (Kumar *et al*. 2010). The primers used in the present study were F-5′-AGAGTTTGATCMTGGCTCA-3′ and R- 5′-TACGGYTACCTTGTTACGACTT -3′. The PCR conditions were the same as those described previously (Kumar *et al*. 2014). The BLAST (blastn) search tool (http://www.ncbi.nlm.nih.gov) was used to analyse the sequences of selected isolates, and the Ez-Taxon server (Chun *et al*. 2007) was used to calculate the pairwise alignment score. The sequences were submitted to GenBank (NCBI). The software contained in the MEGA version 7.0 package was used to conduct phylogenetic and molecular evolutionary analyses (Kumar *et al*. 2016). The 16S rRNA sequences were matched with corresponding nucleotide sequences of bacteria retrieved from various nucleotide databases (GenBank, EMBL, DDBJ, and RDP) using the CLUSTAL W program (Thompson *et al*. 1994). Sequence analysis was performed according to the previous method (Kumar *et al*. 2014).

### Antimicrobial Assay

For the determination of the antimicrobial potential of selected isolated, they were grown in MRS broth (HiMedia, India) at 30°C for incubation for 24 h. The freshly grown isolates were inoculated in the MRS broth and kept at 30°C. After 72 h, these were centrifuged at 8000 rpm for 7–8 min. Sediments were removed after filtration and a microbial filter (syringe filter, HiMedia-SF126, India) is used to filter the fermented samples. Pathogenic bacteria were grown in nutrient agar media (HiMedia, India). The bacterial inoculum of 0.5 McFarland (1.5 × 10^8^ CFU/mL) was prepared. The supernatant (50 μL/well) was checked for antibacterial activity against *S. aureus* MTCC 96, *Bacillus cereus* MTCC 430, *Escherichia coli* MTCC 739, *Salmonella enterica ser. Abony* MTCC3858, *Klebsiella pneumonie* MTCC 4030 because these pathogens are mainly associated with food-borne illness as well drug resistance among these pathogens are also a major concern. The wells were made by using a cork borer (6 mm diameter). In secondary screening, the fermentation conditions and extract preparation were performed according to the method described previously (Gurban oglu Gulahmadov *et al*. 2006). The inhibitory zone diameters were measured after 24-hour incubation period at 37°C.

## RESULTS AND DISCUSSION

### Microbiological Study

A good number of microbes was recorded in the fermented bamboo shoots. *Lactobacillus* species and molds were not recovered in any sample analysed. Most of the strains isolated from the fermented bamboo shoot belong to the genus *Bacillus* and *Enterobacter*. In fermented bamboo shoots, the viable counts ranged from 6.55 ± 0.91 log CFU/g to 7.86 ± 1.21 log CFU/g (Table 1). A total of 10 isolates were selected based on morphology which is designated as DCAST 2, DCAST 3, DCAST 5, DCAST 6, DCAST 8, DCAST 9, DCAST 11, DCAST 14, DCAST 16 and DCAST 17, respectively. These samples were collected from the different districts of Manipur. The highest number of the viable count was found in Churanchandpur (7.86 ± 1.21 log CFU/g), Ukhrul (6.82 ± 1.121 log CFU/g), Noney (6.78 ± 0.23 log CFU/g), etc. and the least amount of viable count was recorded in Senapati (6.55 ± 0.91 log CFU/g) and Thoubal (6.65 ± 0.33 log CFU/g). Based on molecular study all the samples were dominant *Bacillus* except sample 10 (Porompat) in which *Enterobacter* was recovered. The findings of the present study are comparable with those of Khunjan *et al*. (2017). They reported the viable count from 7.47–9.8 log CFU/g of the sample. Sharma and Barooah (2017) reported LAB bacteria in the range of 4.66–7.87 log CFU/g from fermented bamboo shoots.

### Proximate Analysis

Bamboo shoots are rich in various nutrients such as proteins, carbohydrates, vitamins, minerals fibers, phytosterols, phenol and fat (Nirmala *et al*. 2007). There was no significant variability in data generated among all the raw and fermented bamboo shoots in laboratory conditions. The combined proximate chemical composition before and after fermentation including titratable acidity, pH, total phenolic content, ash and reducing sugar is given in Table 2.

### Biochemical Changes by Isolates

After fermentation with isolated microorganisms, the acidity increases as compared to raw bamboo shoots. The increase in fermentation duration is exactly proportional to the drop in pH. The decrease in pH is mainly because of acid production in bamboo shoots (Table 2). The increase in fermentation duration is exactly proportional to the drop in pH. The fermentation of lactic acid in bamboo shoots is mostly responsible for the decrease in pH. The above results are comparable to several FBS products such as *mesu, eup* and *hirring*, which have pH values in the range of 3.9 to 4.1 (Tamang *et al*. 2008; Tamang & Tamang 2009). During the production of Jiang-sun (a Taiwanese FBS product), there was a decrease in pH from 4.2 (First day sample) to 3.5 (30-day sample)(Chen *et al*. 2010). According to previous reports, the pH of hardened tubers of yam (*Dioscorea dumetorum*) fell from 5.5 to 4.8 during the first two days of natural fermentation, and then to 3.9 after 14 days of fermentation (Medoua *et al*. 2008). Acidity was calculated as a percentage of lactic acid. Lactic acid generation during bamboo shoot fermentation could explain the relative rise in high acidity. Fermentation duration had a significant effect on Titratable acidity. *Dioscorea dumetorum* tough tubers showed a similar tendency (Medoua *et al*. 2008). TPC levels increased from 97.5 to 239 mg/100 mL. The level of total phenol increased exponentially as fermentation progressed. In contrast, the TPC of bamboo shoots gradually decreased after storage at 10°C (Badwaik *et al*. 2014). On the other hand, the TPC of control and treated bamboo shoots (salicylic acid) was found to be higher in the treated bamboo shoots. Sugars such as glucose are the best substrate for microbes in the fermentation process. Understanding the dynamics of lowering sugar levels will also aid our understanding of the fermentation process. The amount of reducing sugar is reduced after fermentation.

### Phylogenetic Analysis

The 16s rRNA was submitted to GenBank with accession number given in Table 3. Among all isolates, Isolate number 2 is most closely related to *Bacillus safensis* whose sequence identity diff/total nt is 98.57 (10/700) and the GenBank accession number is MH532552. *B. safensis* is a non-dairy probiotic producing amino acids, vitamins and cofactors. It can tolerate bile salt and exhibit aggregation and adhesion (Saidumohamed & Bhat 2021). The probiotic properties of *B. tequilensis have* been reported previously by Abid *et al*. (2019). Isolate number 3 is most closely related to *B. tequilensis* whose sequence identity diff/total nt is 99.73 (2/753) and the GenBank accession number is MH532553. Isolate number 5 is most closely related to *B. nakamurai* whose sequence identity diff/total nt is 99.48 (4/763) and the GenBank accession number is MH532554. Isolate number 6 is most closely related to *B. safensis* whose sequence identity diff/total nt is 99.47 (4/750) and the GenBank accession number is MH532555 and the isolate number 8 is most closely related to *B. siamensis* whose sequence identity diff/total nt is 100.00 (0/781) and the GenBank accession number is MH532556. Heo *et al*. (2021) reported that *B. siamensis* is a safe strain with the potential to develop as a probiotic.

**Figure 1:**
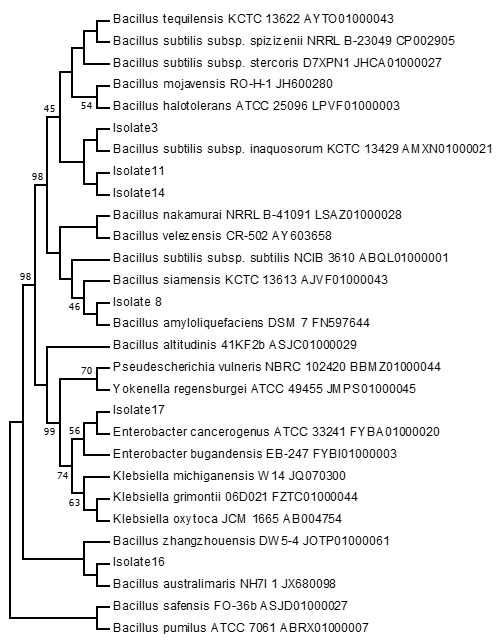
Molecular phylogenetic tree by maximum likelihood method.

**Figure 2:**
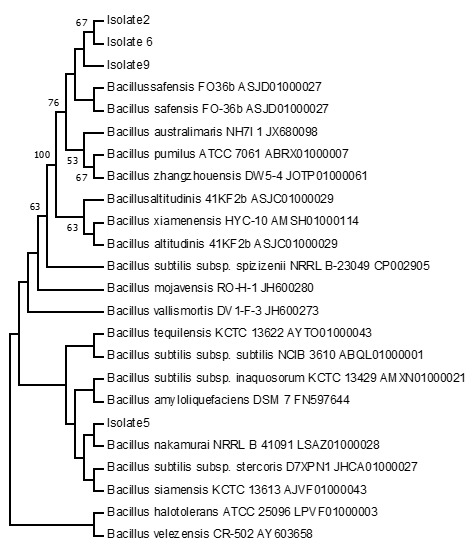
Molecular phylogenetic tree by maximum likelihood method.

The isolate number 9 is most closely related to *B. safensis* whose sequence identity diff/total nt is 98.43 (11/699) and the GenBank accession number is MH532557 and the isolate number 11 is most closely related to *B. subtilis* subsp. *subtilis* whose sequence identity diff/total nt is 99.87 (1/756) and the GenBank accession number is MH532558. Isolate number 14 is most closely related to *Bacillus subtilis* subsp. *stercoris* whose sequence identity diff/total nt is 99.83 (1/594) and the GenBank accession number is MH532559, the isolate number 16 is most closely related to *B. safensis* whose sequence identity diff/total nt is 99.09 (7/771) and the GenBank accession number is MH5325510 and the isolate number 17 is most closely related to *Enterobacter bugandensis* whose sequence identity diff/total nt is 99.8 (1/770) and the GenBank accession number is MH5325511. The phylogenetic analysis revealed that isolate 3 is clustered with *B. subtilis* subsp. *inaquosorum* while isolates 11 and 14 form a new clad (Fig. 1). Therefore, it may belong to the novel species of *Bacillus*. Similarly, isolates 8, 16 and 17 were clustered with *B. amyloliquifaciencs*, *Enterobacter cancerogenus* and *B. australimaris*, respectively. Isolates 2, 6 and 9 form a new clad (Fig. 2) having a boot level confidence of 67% showing that these isolates may belong to novel species. However, to confirm it is novel further studies are required. These findings suggest that fermented bamboo shoots of North East India harbour some novel *Bacillus* species which were not previously reported to date. Jeyaram *et al*. (2010) reported *B. subtilis, B. cereus, L. plantarum* and *Carnobacterium* sp. along with *L. brevis* in soidon by using the molecular technique. In the metagenomic study (fermented bamboo shoots) by Hu *et al*. (2021), the bacteria belonging to 8 phyla, 16 classes, 30 orders, 63 families, 92 genera and 156 species, with *Lactiplantibacillus* accounting for up to 81% of the species, with 12 species, including *L. plantarum*, were reported. This study suggests the huge diversity of bacteria associated with fermented bamboo shoots.

### Detection of Antibacterial Activity

**Figure 3:**
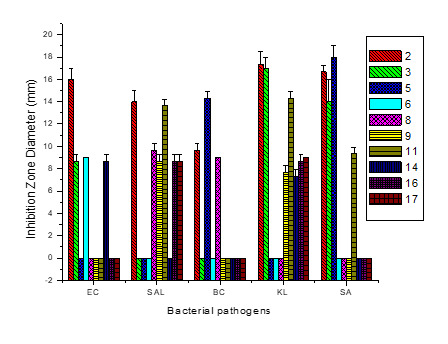
Antibacterial profile of isolates; bar indicates the mean of triplicates ± standard deviation; EC = *E.coli* MTCC 739; SAL = *Salmonella* enterica *ser. Abony* MTCC 3858.; BC = *B. cereus* MTCC 430; KL = *Klebsiella pneumonie* MTCC 4030 ; SA = *S. aureus* MTCC 96.

The emergence of drug resistance among *S. aureus*, *B. cereus*, *E. coli, Salmonella* and *Klebsiella species* is a major concern in the recent era. To find new a chemical entity to combat these pathogens will be an important step in the discovery of new antimicrobials (Founou *et al*. 2016). In this context, the selected isolates were screened for their antibacterial activity against *S. aureus* MTCC 96, *B. cereus* MTCC 430, *E. coli* MTCC 739, *Salmonella enterica ser. Abony* MTCC 3858 and *Klebsiella pneumonie* MTCC 4030. The antibacterial activity of promising isolates is given in Fig. 3. The isolate 2 (*B. safensis*) demonstrated the most promising antibacterial against all the tested bacterial pathogens (Fig. 3). Isolate number 3 (*B. tequilensis*) showed activity against *E. coli, Klebsiella* and *S. aureus*. The isolate 5 (*B. nakamurai*) was effective against*. E. coli*, *Bacillus cereus* and *S. aureus*. The isolates 11 (*B. subtilis* subsp*. subtilis*), 16 (*B. safensis*) and 17 (*E. bugandensis*) demonstrated the most antibacterial intensity to *Salmonella* and *Klebsiella*. While other isolates showed activity against one or more pathogens. As these isolates have not been previously reported from bamboo shoots, hence no comparable data is available.

The production of natural metabolites by probiotics is responsible to repress the growth of both Gram-positive and Gram-negative bacteria. In a study, the bacterial strain *B. tequilensis* was isolated from various traditional Indian fermented products such as fermented batter of Idli, Meduwada and Jalebi and screened its activity against pathogenic *Candida albicans* and non-*albicans* (Palande *et al*. 2015). Thai pickled vegetables (Phak dong) are used as a potential feed supplement in aquaculture. They are mainly used for the growth and disease-resistant alternative for hybrid catfish. *B. siamensis* was isolated from phak dong and it was proved as a probiotic in catfish culture. This bacterium produced bacteriocins-like substances and exhibited a broad-spectrum antibacterial activity (Meidong *et al*. 2017). Among Indonesians, the traditional food “tape” or fermented Cassava is very popular. The quality of this fermented cassava is determined based on the microorganism involved during its presence of fermentation. During the research, 26 amylase-producing *Bacillus* spp. isolates were obtained; among them *B. subtilis* subsp. *subtilis* was dominant. Thus, these isolates can improve the taste and quality of fermented cassava (Barus *et al*. 2013). But none of the isolates of the present study has been previously reported from fermented bamboo shoots except *B. subtilis* and *Enterobacter* sp. (Behera & Balaji 2021).

## CONCLUSION

The present study revealed the presence of *Bacillus safensis, B. tequilensis, B. siamensis, B. nakamurai, B. subtilis* and Enterobacter. These isolates have not been reported previously from fermented bamboo shoots except *B. subtilis and* Enterobacter. Interestingly, no *Lactobacillus* species and molds were not detected in any of the analysed samples. Potent antibacterial activity was recorded against *Klebsiella*, *Staphylococcus aureus*, *Salmonella* and *Bacillus cereus*. The results of the present study are promising however, further studies are required to isolate, purify and characterise the metabolites produced by these isolates.

## Figures and Tables

**Table 1:** Viable count of probiotic bacteria isolated from different fermented foods.

Sample no.	Place	Viable count Log CFU/g	No. of isolates selected based on morphology	Coding
1	Bishnupur	6.65 ± 0.41	01	DCAST2
2	Thoubal	6.65 ± 0.33	01	DCAST3
3	Senapati	6.55 ± 0.91	01	DCAST5
4	Ukhrul	6.82 ± 1.121	01	DCAST6
5	Chandel	6.74 ± 0.52	01	DCAST8
6	Jiribam	6.68 ± 0.12	01	DCAST9
7	Churachandpur	7.86 ± 1.21	01	DCAST11
8	Kakching	6.76 ± 0.43	01	DCAST14
9	Noney	6.78 ± 0.23	01	DCAST16
10	Porompat	6.85 ± 0.84	01	DCAST17

**Table 2:** Fermented bamboo shoot sample: influence of fermentation (at 25°C–27°C for two months) on the raw and fermented shoot.

Characteristics	Raw bamboo shoots	Fermented bamboo shoot
Titratable acidity	0.89 ± 0.01	4.09 ± 0.01
pH	6.74 ± 1.75	5.40 ± 0.40
Moisture content	68.42 ± 2.02	91.12 ± 3.68
Total phenolic content (mg/100 g)	97.50 ± 1.20	239.00 ± 1.00
Ash	0.86 ± 0.00	0.81 ± 0.01
Reducing sugar (g/100 g)	1.37 ± 0.01	0.26 ± 0.01

*Note*: Values are average of 10 samples ± standard deviation

**Table 3:** GenBank accession number, sequence identity and most closet neighbours of most promising isolates.

Selected isolates	GenBank accession no.	Sequence identity diff/total nt	Most closely related with
Isolate 2	MH532552	98.57 (10/700)	*B. safensis*
Isolate 3	MH532553	99.73 (2/753)	*B. tequilensis*
Isolate 5	MH532554	99.48 (4/763)	*B. nakamurai*
Isolate 6	MH532555	99.47 (4/750)	*B. safensis*
Isolate 8	MH532556	100.00 (0/781)	*B. siamensis*
Isolate 9	MH532557	98.43 (11/699)	*B. safensis*
Isolate 11	MH532558	99.87 (1/756)	*B. subtilis* subsp. *subtilis*
Isolate 14	MH532559	99.83 (1/594)	*B. subtilis* subsp. *stercoris*
Isolate 16	MH5325510	99.09 (7/771)	*B. safensis*
Isolate 17	MH5325511	99.8 (1/770)	*Enterobacter bugandensis*

## References

[b1-tlsr-33-3-151] Abid Y, Azabou S, Casillo A, Gharsallah H, Jemil N, Lanzetta R, Attia H, Corsaro MM (2019). Isolation and structural characterization of levan produced by probiotic Bacillus tequilensis-GM from Tunisian fermented goat milk. International Journal of Biological Macromolecules.

[b2-tlsr-33-3-151] AOAC (1990). Approved methods of Association of Official Analytical Chemists.

[b3-tlsr-33-3-151] Badwaik LS, Borah PK, Borah K, Das AJ, Deka SC, Sharma HK (2014). Influence of fermentation on nutritional compositions, antioxidant activity, total phenolic and microbial load of bamboo shoot. Food Science and Technology Research.

[b4-tlsr-33-3-151] Barus T, Kristani A, Yulandi A (2013). Diversity of amylase-producing *Bacillus* spp. from “tape”(fermented *Cassava*). HAYATI Journal of Biosciences.

[b5-tlsr-33-3-151] Behera P, Balaji S (2021). Health benefits of fermented bamboo shoots: The twenty-first century green gold of northeast India. Applied Biochemistry and Biotechnology.

[b6-tlsr-33-3-151] Bray HG, Thorpe WV (1954). Analysis of phenolic compounds of interest in metabolism. Methods of Biochemical Analysis.

[b7-tlsr-33-3-151] Chen YS, Wu HC, Liu CH, Chen HC, Yanagida F (2010). Isolation and characterization of lactic acid bacteria from jiang-sun (fermented bamboo shoots), traditional fermented food in Taiwan. Journal of the Science of Food and Agriculture.

[b8-tlsr-33-3-151] Choudhury D, Sahu JK, Sharma GD (2012). Value addition to bamboo shoots: A review. Journal of Food Science and Technology.

[b9-tlsr-33-3-151] Chun J, Lee JH, Jung Y, Kim M, Kim S, Kim BK, Lim YW (2007). EzTaxon: A web-based tool for the identification of prokaryotes based on 16S ribosomal RNA gene sequences. International Journal Systematic and Evolutionary and Microbiology.

[b10-tlsr-33-3-151] Founou LL, Founou RC, Essack SY (2016). Antibiotic resistance in the food chain: A developing country perspective. Frontiers in Microbiology.

[b11-tlsr-33-3-151] Gandhi YS, Bankar VH, Vishwakarma RP, Satpute SR, Upkare MM (2017). Reducing sugar determination of jaggery by classical lane and eynon method and 3, 5-Dinitrosalicylic acid method. Imperial Journal of Interdisciplinary Research.

[b12-tlsr-33-3-151] Goyal AK, Middha SK, Usha T, Chatterjee S, Bothra AK, Nagaveni MB, Sen A (2010). Bamboo-infoline: A database for North Bengal bamboo’s. Bioinformation.

[b13-tlsr-33-3-151] Gurban oglu Gulahmadov S, Batdorj B, Dalgalarrondo M, Chobert JM, Alekper oglu Kuliev A, Haertlé T (2006). Characterization of bacteriocin-like inhibitory substances (BLIS) from lactic acid bacteria isolated from traditional Azerbaijani cheeses. European Food Research and Technology.

[b14-tlsr-33-3-151] Heo SJ, Kim JH, Kwak MS, Jeong DW, Sung MH (2021). Functional genomic insights into probiotic *Bacillus siamensis* strain B28 from traditional korean fermented kimchi. Foods.

[b15-tlsr-33-3-151] Hu Y, Chen X, Zhou J, Jing W, Guo Q (2021). Metagenomic analysis of suansun, a traditional chinese unsalted fermented food. Processes.

[b16-tlsr-33-3-151] Isolauri E, Salminen S, Ouwehand AC (2004). Probiotics. Best Practice and Research Clinical Gastroenterology.

[b17-tlsr-33-3-151] Jeyaram K, Romi W, Singh TA, Devi AR, Devi SS (2010). Bacterial species associated with traditional starter cultures used for fermented bamboo shoot production in Manipur state of India. International Journal of Food Microbiology.

[b18-tlsr-33-3-151] Khunjan O, Pandey P, Sharma GD (2017). Microbial fermentation by traditional process using intrinsic microflora reduces the cyanide content of bamboo shoots. Journal of Pure and Applied Microbiology.

[b19-tlsr-33-3-151] Kumar S, Stecher G, Tamura K (2016). MEGA7: Molecular evolutionary genetics analysis version 7.0 for bigger datasets. Molecular Biology and Evolution.

[b20-tlsr-33-3-151] Kumar V, Bharti A, Gusain OP, Bisht GS (2010). An improved method for isolation of genomic DNA from filamentous actinomycetes. Journal of Science, Engineering and Technology Management.

[b21-tlsr-33-3-151] Kumar V, Naik B, Gusain O, Bisht GS (2014). An actinomycete isolate from solitary wasp mud nest having strong antibacterial activity and kills the *Candida* cells due to the shrinkage and the cytosolic loss. Frontiers in Microbiology.

[b22-tlsr-33-3-151] Medoua GN, Mbome IL, Agbor-Egbe T, Mbofung CMF (2008). Influence of fermentation on some quality characteristics of trifoliate yam (*Dioscorea dumetorum*) hardened tubers. Food Chemistry.

[b23-tlsr-33-3-151] Meidong R, Doolgindachbaporn S, Jamjan W, Sakai K, Tashiro Y, Okugawa Y, Tongpim S (2017). A novel probiotic *Bacillussiamensis* B44v isolated from Thai pickled vegetables (Phak-dong) for potential use as a feed supplement in aquaculture. Journal of General and Applied Microbiology.

[b24-tlsr-33-3-151] Melini F, Melini V, Luziatelli F, Ficca AG, Ruzzi M (2019). Health-promoting components in fermented foods: An up-to-date systematic review. Nutrients.

[b25-tlsr-33-3-151] Nirmala C, David E, Sharma ML (2007). Changes in nutrient components during ageing of emerging juvenile bamboo shoots. International Journal of Food Sciences and Nutrition.

[b26-tlsr-33-3-151] Palande V, Meora R, Sonavale RM, Makashir M, Modak MS, Kapse N, Dhakephalkar PK, Ranjekar PK, Bipinraj Nirichan Kunchiraman BN (2015). Inhibition of pathogenic strains of *Candida albicans* and non-albicans by *Bacillus* species isolated from traditional Indian fermented food preparations. International Journal of Current Microbiology and Applied Sciences.

[b27-tlsr-33-3-151] Saidumohamed BE, Bhat SG (2021). Indian oil sardine (*Sardinella longiceps*) gut derived *Bacillus safensis* SDG14 with enhanced probiotic competence for food and feed applications. Food Research International.

[b28-tlsr-33-3-151] Sharma N, Barooah M (2017). Microbiology of Khorisa, its proximate composition and probiotic potential of lactic acid bacteria present in Khorisa, a traditional fermented bamboo shoot product of Assam. Indian Journal of Natural Products and Resources.

[b29-tlsr-33-3-151] Tamang B, Tamang JP (2009). Lactic acid bacteria isolated from indigenous fermented bamboo products of Arunachal Pradesh in India and their functionality. Food Biotechnology.

[b30-tlsr-33-3-151] Tamang B, Tamang JP, Schillinger U, Franz CM, Gores M, Holzapfel WH (2008). Phenotypic and genotypic identification of lactic acid bacteria isolated from ethnic fermented bamboo tender shoots of North East India. International Journal of Food Microbiology.

[b31-tlsr-33-3-151] Tamang JP, Shin DH, Jung SJ, Chae SW (2016). Functional properties of microorganisms in fermented foods. Frontiers in Microbiology.

[b32-tlsr-33-3-151] Tamang JP, Tamang JP, Kailasapathy K (2010). Diversity of fermented foods. Fermented foods and beverages of the world.

[b33-tlsr-33-3-151] Thakur K, Rajani CS, Tomar SK, Panmei A (2016). Fermented bamboo shoots: A riche niche for beneficial microbes. Journal of Bacteriology and Mycology.

[b34-tlsr-33-3-151] Thompson JD, Higgins DG, Gibson TJ (1994). CLUSTAL W: Improving the sensitivity of progressive multiple sequence alignment through sequence weighting, position-specific gap penalties and weight matrix choice. Nucleic Acids Research.

[b35-tlsr-33-3-151] Tindall BJ, Rosselló-Móra R, Busse HJ, Ludwig W, Kämpfer P (2010). Notes on the characterization of prokaryote strains for taxonomic purposes. International Journal of Systematic and Evolutionary Microbiology.

